# Theoretical and Experimental Investigations of the Potential of Osmotic Energy for Power Production †

**DOI:** 10.3390/membranes4030447

**Published:** 2014-08-08

**Authors:** Adel O. Sharif, Ali A. Merdaw, Maryam Aryafar, Peter Nicoll

**Affiliations:** 1 Centre for Osmosis Research and Applications, Chemical & Process Engineering Department, University of Surrey, Guildford GU2 7XH, UK; E-Mails: alimerdaw@yahoo.com (A.A.M.); m.aryafar@surrey.ac.uk (M.A.); 2 Qatar Energy and Environment Research Institute, Qatar Foundation, Doha 5825, Qatar; 3 Modern Water plc, Guildford GU3 1LR, UK; E-Mail: peter.nicoll@modernwater.co.uk

**Keywords:** pressure retarded osmosis, osmotic energy, hydro-osmotic power (HOP), forward osmosis, specific energy consumption (SEC)

## Abstract

This paper presents a study on the potential of osmotic energy for power production. The study includes both pilot plant testing and theoretical modelling as well as cost estimation. A projected cost of £30/MWh of clean electricity could be achieved by using a Hydro-Osmotic Power (HOP) plant if a suitable membrane is used and the osmotic potential difference between the two solutions is greater than 25 bar; a condition that can be readily found in many sites around the world. Results have shown that the membrane system accounts for 50%–80% of the HOP plant cost depending on the salinity difference level. Thus, further development in membrane technology and identifying suitable membranes would have a significant impact on the feasibility of the process and the route to market. As the membrane permeability determines the HOP process feasibility, this paper also describes the effect of the interaction between the fluid and the membrane on the system permeability. It has been shown that both the fluid physical properties as well as the membrane micro-structural parameters need to be considered if further development of the HOP process is to be achieved.

## 1. Introduction

**Figure 1:**
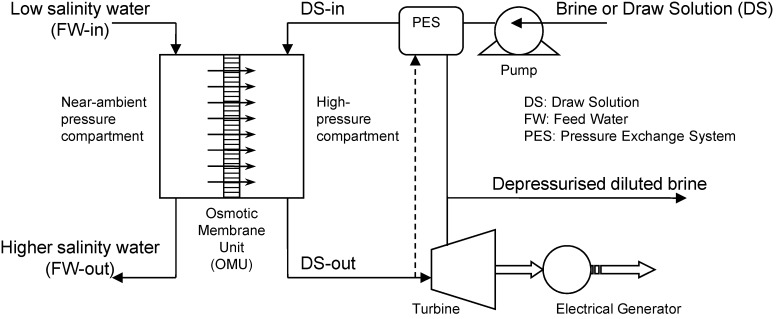
Schematic diagram of the open-cycle Hydro-Osmotic Power (HOP) plant.

The world’s search for cost-effective Renewable Energy (RE) sources is continuous and has taken many dimensions and directions. This has become more so, given the current urgency of climate change, dwindling world supplies of conventional fossil fuels, and increasing oil prices. Alternative energy sources, including solar, wind, tidal wave, and biomass, have been used to provide secure, sustainable and adequate energy sources. However, expensive equipment and high installation costs of these technologies, coupled with the uneven availability distribution, have prevented them, so far, from being used widely [1]. Recently, osmotic energy has been introduced as a source of renewable and sustainable energy, and it shows potential for power production. Osmotic Energy (OE) is released in the process of mixing a low concentration solution, which has a relatively low osmotic pressure, and a high concentration solution, which normally has a higher osmotic pressure, through a semi-permeable membrane. The membrane retains the solute movement between the two solutions and only allows pure water to pass. This can be achieved by using fresh water, brackish water or waste water effluent as the lower osmotic potential side and a saltier water such as seawater or brine as the high osmotic potential side to create the required osmotic pressure difference to run the process. This osmotic energy can be converted into mechanical energy through a Pressure Retarded Osmosis (PRO) process and recovered as hydropower [2,3]. The recovered pressure can be used to generate electricity using a hydro-turbine and generator in the form of a land based Hydro-Osmotic Power (HOP) plant [4,5,6]. There are a number of ways to recover the OE of concentrated salty solutions including open cycle and closed cycle HOP systems. The principle of an open cycle system (Figure 1) is that low salinity water, Feed Water (FW), is fed at low osmotic and hydraulic pressures to one side of an Osmotic Membrane Unit (OMU), while a Draw Solution (DS), e.g., seawater or brine, is fed to the other side at higher osmotic and hydraulic pressures. The hydraulic pressure of the DS is normally less than the osmotic pressure. The diluted DS is used to operate a turbine in order to generate power. A more efficient process can be achieved by recycling some of the pressurized solution, leaving the OMU, and through a pressure exchange system (PES) to assist in pumping the brine to the OMU. This process is applied when there is a continuous supply of freshwater and seawater, e.g., at a river run-off point to a sea or to a salty lake [7].

**Figure 2:**
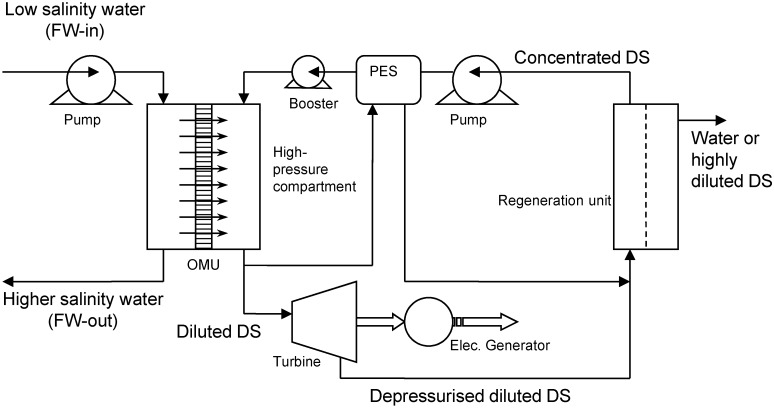
Schematic diagram of the closed cycle HOP plant.

Alternatively, in a closed cycle HOP plant [8], which is schematically shown in Figure 2, the DS is retained in the system by using a Regeneration Unit (RU) such as evaporation, crystallization, membrane separation, or other solutes concentration techniques. In the closed cycle HOP plant, the generated hydraulic pressure can be used to produce electricity in a similar way to the open cycle system or could be transferred to other liquids through a PES for pumping processes. The efficiency of the closed HOP system depends on the availability of a low-grade energy and/or renewable energy sources for the regeneration of the osmotic agents [9]. Examples of renewable energy sources include solar, geothermal, and wind for evaporation in hot and dry climates or cold temperature for crystallization in cold climates, and/or waste heat from power and chemical plants anywhere.

Since PRO inception in 1954 [2], the concept of osmotic energy recovery has received sporadic attention, mainly in the form of design studies and preliminary economic viability evaluations. A review of published material and experimental data on pressure retarded osmosis (PRO) are given in Table 1 including the characteristics of commercial and prototype osmotic membranes and the power density through PRO processes in different investigations.

**Table 1:** Review of Pressure Retarded Osmosis (PRO) studies from the early days to the most recent Investigations [10]. Data from references [7,10,11,12,13,14,15,16,17,18,19,20,21,22,23,24,25,26,27].

Researcher (Year)	Feed/Draw Solution	Membrane	Hydraulic Pressure (bar)	Power Density (W/m^2^)	Ref.
Loeb *et al.* (1976)	Freshwater/Seawater	Hollow Fibre seawater RO	12	0.35	[11]
Mehta (1978)	Freshwater/Brine	Hollow Fibre seawater RO	40	3.27	[12]
Loeb & Mehta (1979)	Freshwater/Brine	FRL Composite seawater RO	19	1.56	[13]
Mehta & Loeb (1979)	Freshwater/Brine	Hollow Fibre seawater RO	40	3.12	[14]
Lee *et al.* (1980)	Freshwater/Brine (3.5%)	CA& PA& PBIL flat sheet seawater RO	12.5	1.55	[15]
Jellinek & Masuda (1981)	Freshwater/Brine	seawater spiral wound RO	11–16	1.6	[16]
Gerstandt *et al.* (2008)	Freshwater/Seawater	Lab TFC (flat sheet/hollow fibre) and CA	10–13	1.3–3.5	[17]
Skilhagen *et al.* (2008)	Freshwater/Seawater	Modified TFC for PRO	11–15	1.0	[7]
Achilli *et al.* (2009)	DI water/Brine (3.5%–6%)	CTA flat sheet seawater FO (HTI)	9.7	2.7–5.1	[18]
Thorsen & Holt (2009)	Freshwater/Seawater	TFC & CTA commercial FO flat sheet seawater	7–12	1.6–2.7	[19]
Chou *et al.* (2011)	River water & waste water/Brine (3.5%–6%)	TFC Hollow Fibre seawater FO	8–9.1	8.4–11	[20]
Yip & Elimelech (2011)	River water/Seawater (3.5%)	Modified TFC membrane, Hollow Fibre	10–15	6.1–10	[21]
She *et al.* (2012)	Freshwater/Brine (2 M NaCl)	CTA commercial flat sheet seawater FO(HTI)	13	6.7	[22]
Kim & Elimelech (2012)	NaCl solution (0.5–1 M)/Brine (2 M)	CTA commercial FO flat sheet	12.5	4.7	[23]
Wang *et al.* (2012)	River & Wastewater (0.5 M)/Seawater	TFC hollow fibre commercial FO	5–8.9	4.1–5.7	[24]
Schiestel *et al.* (2012)	Freshwater/Seawater	CTA commercial FO flat sheet	8	2.25	[25]
Saito *et al.* (2012)	Waste water/RO Brine (2–2.5 M)	commercial FO hollow fibre	25	7.7	[26]
Efraty (2012)	Freshwater/Seawater	Modified TFC membrane	9.6	7.4	[27]

The majority of the mentioned PRO studies in the literature have focused on fresh water/seawater or brine resources in HOP system. The results show the high potential of the PRO system for power production. For instance, each cubic meter of saltwater (salinity of 3.5%) has, in theory, 0.7 kWh/m^3^ of energy [28]. However, for higher salinity solutions, such as the Dead Sea (33.7% salinity) or other salty lakes, for example Sawa Lake in Iraq (~15.8% salinity), the potential of electrical energy production of each cubic meter could be greater than 5 kWh/m^3^ [29]. However, this potential of the OE is limited by water flux rate and the permeability of the membrane. Though higher osmotic pressure difference increases the flux, previous studies have shown that increasing the solute concentration gradient across the membrane reduces the membrane permeability [6] and that in this case, a suitable membrane is the most important component of the osmotic power plant [7].

In this paper, a theoretical study of the potential of osmotic energy for power generation is introduced based on a salinity gradient for PRO power generation using the Solution-Diffusion Pore-Flow Fluid-Resistance (SDPFFR) model. Furthermore, the potential of PRO osmotic power generation is investigated via the hybrid FO-RO pilot plant performance experiments using two available commercial membranes. Finally, the cost of electricity of a closed cycle HOP plant for 25 MW net power production is estimated by utilizing 15 bar hydraulic pressure at the DS side.

## 2. Basic Theory

Water flux across the membrane in PRO processes, *J_w_*, is usually represented by the following phenomenological relationship:

*J_w_* = *A_w_* (∆П − ∆*P*) (1)

Equation (1) shows that *J_w_* is determined as the product of the system permeability to water, *A_w_*, and the net trans-membrane driving pressure, which is the net difference between the osmotic pressure, ∆П, and the net hydraulic pressure, ∆*P*. The density of the power obtained from the PRO process, *W*, can be estimated as the product from multiplying the water flux by the hydraulic pressure [17,18]:

*W* = *J_w_* ∆*P* = *A_w_* (∆П − ∆*P*)∆*P* (2)

The power density of the membrane that is required to obtain a profitable PRO process was determined to be between 4 and 6 W/m^2^ [17]. A suggestion was made by another study [18] that the first derivative for Equation (2) with respect to ∆*P* assuming *A_w_* as a constant may specify the maximum value for *W*:

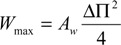
(3)


**Figure 3:**
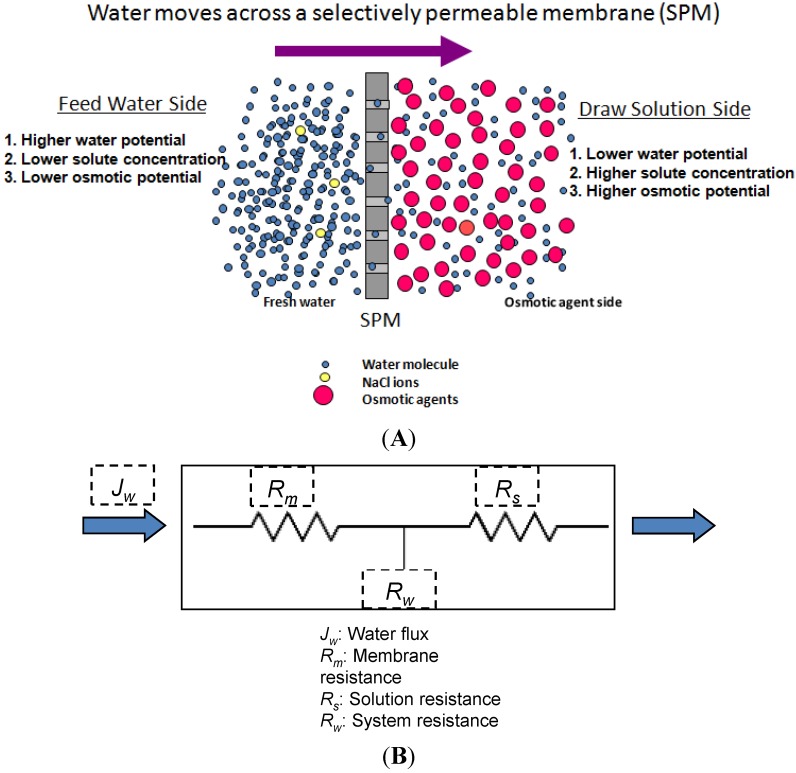
(**A**) Schematic representation for water flux and solute concentration across a pore of a membrane in the PRO process; (**B**) electrical analogy for the system resistance to water flux.

Hence, from Equation (2), *W_max_* can be calculated, which can be reached when ∆*P* equals 0.5∆П. The ∆*P* is normally estimated by assuming linear pressure drop alongside the OMU, *i.e.*,

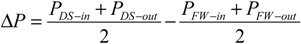
(4)
and similarly for ∆П:

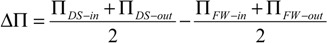
(5)
Where, the subscripts DS and FW refer to the Draw Solution (the high concentration side) and the Feed Water (the low concentration side), respectively, as illustrated in Figure 1. As mentioned earlier, it assumes that *A_w_* is constant. It also assumes that the osmotic pressure of the particles dissolved within the liquid is equal to the ideal gas pressure in the gas phase, *i.e.*, Π = *P* [30]. Actually, *A_w_* is a variable parameter that depends on the process conditions; it decreases with ∆*P* or ∆П increase, and increases with temperature rise. Moreover, the measured value of the osmotic pressure, П, at the bulk solution is not necessarily the same at the membrane surface or does not have a similar effect for a similar value of hydraulic pressure, *P*. Therefore, many correlations have been suggested in literature to allow Equation (1) to represent the actual conditions by, e.g., adding a membrane reflection coefficient or a concentration polarization modulus. It is suggested that *A_w_* can be empirically described using the Solution-Diffusion Pore-Flow Fluid-Resistance (SDPFFR) model [31,32], as illustrated in Figure 3. According to this model, the total system resistance to water transfer is a sum of two resistances connected in series, membrane material resistance and solution resistance. Hence the system permeability, *A_w_*, which is the reciprocal of the system resistance, is estimated as a product from the interaction between the membrane phase permeability, *A_wm_*, and the solution phase, the DS and the FW, permeability, *A_ws_*:

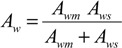
(6)


From which water flux is obtained as follows:

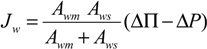
(7)


The *A_wm_*, depends on the membrane intrinsic microstructure and the physical properties of the pure water, while the *A_ws_* is controlled by the solution molecular and the physical properties.

In most cases with FO and PRO processes, the *A_wm_*, is considered to be a constant value due to the negligible effect of the low hydraulic pressure on membrane microstructure. This permeability coefficient can be experimentally estimated by carrying out an RO experiment at the ∆*P* value of interest with pure water as feed. It can also be analytically determined according to the Analytical Solution-Diffusion Pore-Flow (ASDPF) model [33], which assumes that pure water flux across the membrane is jointly carried out by diffusion and pore flow mechanisms:

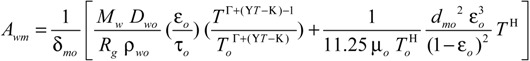
(8)


The δ*_mo_*, ε*_o_*, τ*_o_*, and *d_mo_* are the membrane thickness, the porosity, the tortuosity, and the mean pore diameter, respectively. The *D_wo_*, ρ*_wo_*, μ*_o_* and *M_w_* are the self-diffusivity, the density, the viscosity, and the molecular weight of pure water at the reference temperature (*T_o_* = 298.15 K), while *R_g_* is the universal gas constant. The parameters *T*, *Y*, *K*, and *H* are the constant values 6.52222, 0.00145, 0.35748, and 6.81324, respectively. The effect of temperature on *A_wm_* can be indicated from Equation (8); it is attributed to the fluid properties of water. However, it has experimentally been shown that *A_wm_* increases in a power function with temperature [34]:

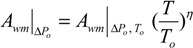
(9)


The *A_ws_* represents the ability of water to pass through solute molecules or particles that obstruct it and move in an opposite direction. This permeability is affected by several parameters depending on the solute-solvent interactions and the solution-membrane interactions. The membrane structure may interact with the nearby ions and affects their movement, which correspondingly affects the solution permeability. The *A_ws_* by this definition accounts for all the effects on water flux that may occur in the solution outside the membrane (e.g., the concentration polarization layer) and inside the membrane (e.g., the electrostatic effects of the structure). Experimentally, *A_ws_* can be estimated from the observed system permeability and the measured or the calculated membrane permeability as follows:

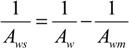
(10)


**Figure 4:**
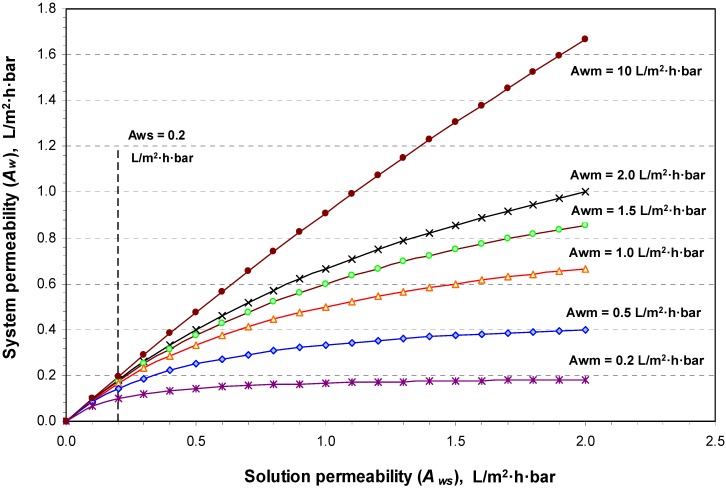
The system permeability (*A_w_*) in membrane separation processes as a function of the solution permeability (*A_ws_*) at different values for the membrane permeability (*A_wm_*).

By carrying out a PRO experiment, the value of *A_ws_* can be determined as a function of any of the operational conditions. From a comparison between the obtained values for *A_wm_* and *A_ws_* in each case, the dominant resistance to water flux can be determined. This mathematical representation for the interaction between the membrane and the solution indicates that *A_wm_* and *A_ws_* equally influence the overall system permeability, *A_w_*, which is the critical process parameter. Hence, any future efforts sought to develop this process should consider system phases, the membrane, and the solution. Improvements to membrane permeability only by e.g., increasing the porosity or reducing the thickness, would not necessarily lead to an increase of the system permeability if the draw solution properties are not considered. Figure 4, which demonstrates the theoretical relationship (10), shows that increasing *A_wm_* 10 times from 0.2 to 2.0 L/m^2^·h·bar will increase *A_w_* from 0.1 to only 0.18 L/m^2^·h·bar if *A_ws_* remains constant at 0.2 L/m^2^·h·bar, and if *A_wm_*, for example, increased further 50 times to 10 L/m^2^·h·bar, *A_w_* will not increase to more than 0.2 L/m^2^·h·bar.

The *A_ws_* is reversibly proportional to the concentration difference across the membrane. It has two limits, the highest when *A_ws_* → ∞ for pure solvents, and the lowest when *A_ws_* → 0 as the concentration of the solute approaches saturation. However, the saturation point cannot be regarded as an infinite concentration as the process is dynamic and water continuously transfers across the membrane diluting the DS. Hence, the value of *A_ws_* never actually reaches zero. In small size applications, such as the bench-scale experiments, the DS concentration decreases and the FW concentration increases with time. In commercial modules, where cross flow regimes are normally utilized, the concentrations change with membrane length. In processes that utilize co-current cross-flow modes, the lowest solution permeability may occur at the concentrated DS inlet position where the FW enters at its lowest concentration. As the concentration difference across the membrane continuously decreases, the solution permeability to transfer water continuously increases. Finally, many practical parameters and indices can be identified for the PRO process in an open HOP plant. A material balance around the OMU gives the following:

*Q_DS−out_* − *Q_DS−in_* = *Q_w_* = *Q_FW−in_* − *Q_FW−out_* (11)

Where *Q* is the volumetric flow rate and *Q_w_* refers to the permeated water flow rate across the membrane. The gross power production, *P_G_*, that could be produced by a PRO process can be mathematically represented using the following relationship:

*P_G_* = *P_DS−out_* ∙ *Q_DS−out_* − *P_DS−in_* ∙ *Q_DS−in_* (12)

The energy density, ρ*_E_*, of the inlet DS can be represented as:

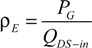
(13)


The specific energy production, *E_S_*, can be calculated as:

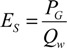
(14)


## 3. Experimental Setup

**Figure 5:**
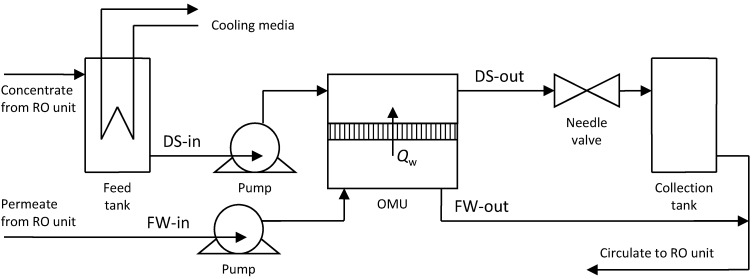
Schematic diagram for the pilot plant setup.

The pilot plant setup is schematically shown in Figure 5. A controlling needle valve was used to represent the turbine generator assembly. The OMU module used had a high surface area of around 100 m^2^. The OMU discharged streams were circulated to an RO unit to regenerate the DS as well as the diluted FW. A cooling system for the feed tank was used to prevent temperature increase during operation. The inlet FW was fed to the module at constant hydraulic pressure, though its flow rate was variable depending on the rate of membrane flux. The concentration measurements at the different locations of the plant were obtained by using a portable conductivity meter, while flow rate and pressure measurements were taken from inline digital indicators. Two flat sheet membranes TFC-ULP and TFC-HP with polyamide salt-rejection film produced by Koch membrane Systems were tested in this study.

The DS and FW used were aqueous solutions of NaCl salt at different concentrations to simulate fresh water (280 ppm), brackish water (6900 ppm), seawater (~35,000 ppm), and brine (145,000 ppm). Table 2 shows the main operational conditions of three selected experiments. Several pilot plant runs were carried out under variable DS inlet hydraulic pressures at a constant temperature of 25 °C and specific feed flow rates.

**Table 2:** The operational conditions for the pilot plant runs.

Experiment No.	FW-in		DS-in		Osmotic pressure difference ∆Π_f_, bar
	Concentration, ppm	Flow Rate, L/min	Concentration, ppm	Flow Rate, L/min	
1	240	11.1	34,560	9.8	27.4
2	6900	10.9	145,000	5.5	87.3
3	6900	9.5	34,690	5.5	22.1

## 4. Results and Discussion

### 4.1. Theoretical Calculation Results

A closed-cycle HOP was foreseen for 25 MW net electricity production, as shown in Figure 2. The several designs parameters were assumed to carry out the calculations; these are listed in Table 3.

**Table 3:** Estimated input design parameters.

**Osmotic membrane unit (OMU)**	**Value**
Inlet (concentrated) DS hydraulic pressure (bar)	15.0
DS side hydraulic pressure drop (%)	10
Membrane element area (m^2^)	100.8
DS dilution rate (%)	100
Freshwater inlet hydraulic pressure (bar)	0.4
Freshwater inlet osmotic pressure (bar)	0.0
Discharged freshwater hydraulic pressure (bar)	0.1
Freshwater recovery rate (%)	95
**Regeneration unit (RU) (FO unit)**	**Value**
Brine inlet maximum osmotic pressure (bar)	250
Brine inlet hydraulic pressure (bar)	2.0
Discharged (diluted) brine hydraulic pressure (bar)	0.1
DS side hydraulic pressure drop (%)	30
DS recovery rate (%)	50
Brine dilution rate (%)	100
Membrane element area (m^2^)	100
**Efficiencies**	**Value**
Pumps and booster (%)	80
Turbine and generator (%)	80
PES (%)	95
Diluted DS (PES/Turbine) input flowrate ratio	1.5

**Figure 6:**
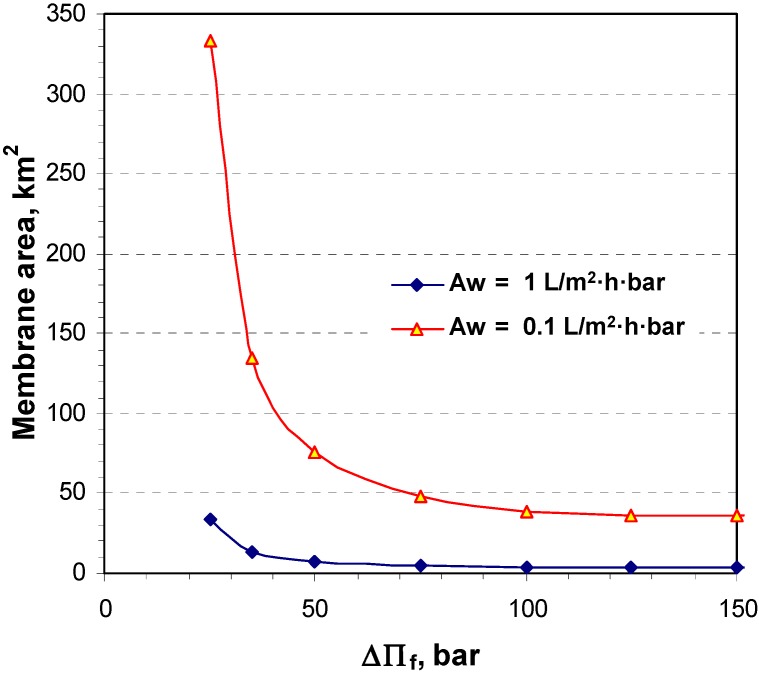
The estimated total membrane area of a closed cycle HOP plant for 25 MW net power production as a function of the osmotic pressure differnces (**∆**П_f_) at the Osmotic Membrane Unit (OMU) utilizing 15 bar hydraulic pressure at the Draw Solution (DS) side.

For two different system permeabilities (*A_w_*), 0.1 and 1 L/m^2^·h·bar, the total required membrane area as a function of the osmotic pressure differences, ∆П_f_, between the inlet concentrated DS and the inlet diluted FW at the OMU is shown in Figure 6.

The results indicate that a substantial reduction in the membrane area requirement for a given power output can be achieved as the osmotic pressure difference increases beyond 50 bar.

**Figure 7:**
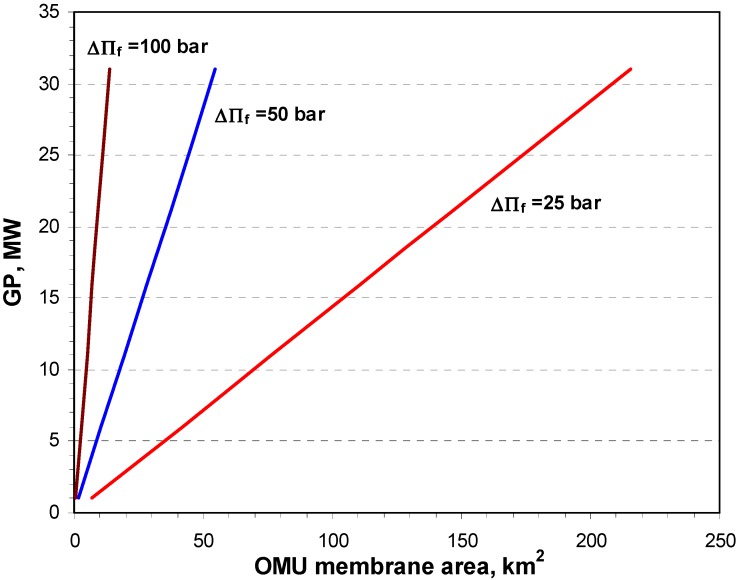
Gross power produced (*P_G_*) as a function of the OMU membrane area. (The DS side hydraulic pressure in each case is set to be equal to 0.5**∆**H_f_, and the system permeability is assumed to be 0.1 L/m^2^·h·bar).

Furthermore, independent of the membrane permeability, based on the same above-mentioned assumptions but with an inlet (concentrated) DS hydraulic pressure equal to 50% of the **∆**П_f_, the produced gross power *P_G_* was calculated, as illustrated in Figure 7, as a function of the estimated OMU membrane area assuming 0.1 L/m^2^·h·bar permeability for different values of **∆**П_f_.

As can be seen in Figure 7, the gross power increases almost linearly with the increase in OMU membrane area because the enhanced water flux for a given membrane permeability. However the rise of gross power caused by increasing the osmotic pressure difference is severe in the low membrane area range. According to the predicted gross power per membrane area illustrated by the power density (W/m^2^) in Figure 7, the osmotic pressure difference of more than 50 bar is preferred as it allows the generation of a higher power density requiring a lower membrane area for the given 0.1 L/m^2^·h membrane permeability. Therefore, the development of new membranes with high permeability and the ability to endure high pressure differences is essential for achieving maximum power density and, consequently, marketable energy cost.

### 4.2. Pilot Plan Performance Test Results

The membrane permeability (*A_wm_*) was firstly measured by using pure water as feed at 25 °C. The test was carried out by modifying the OMU to an RO setup. The *A_wm_* was found to decrease with ∆*P* within an experimental range of 5 to 30 bar, according to the following relationship, in L/m^2^·h·bar:

*A_w_* = 0.3265 − 0.045ln(∆*P*) (15)

**Figure 8:**
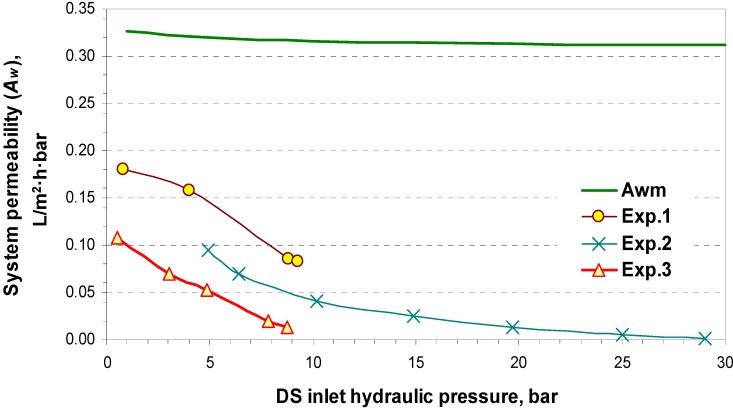
The system permeability coefficient (*A_w_*) as a function of the DS inlet hydraulic pressure. (The numbers 1, 2, and 3 on the graph refer to the experiment number).

The system permeability (*A_w_*) was then experimentally determined in a PRO setup as the product from dividing the measured water flux by the net driving pressure (∆П–∆*P*). The obtained values for *A_w_* are plotted in Figure 8 as a function of the DS inlet hydraulic pressure. Each experiment is referred to by its number in Table 2. The *A_wm_* is also shown in Figure 8 for comparison.

**Figure 9:**
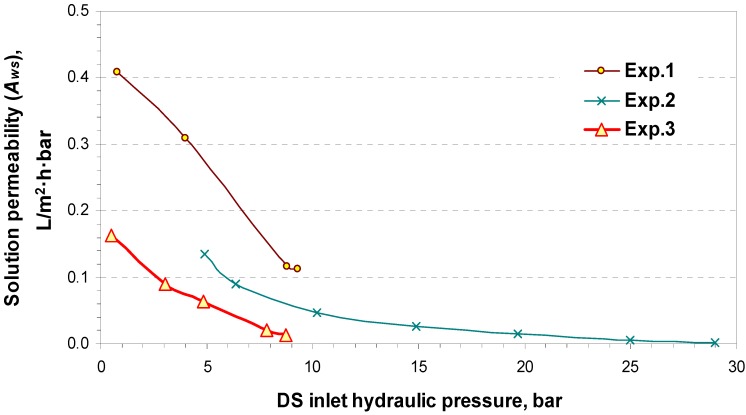
The solution permeability coefficient (*A_ws_*) as a function of the DS inlet hydraulic pressure.

The *A_wm_* is the upper limit for the *A_w_*; as the concentration of the entered solutions or the ∆*P* increases, *A_w_* differs from *A_wm_*. This indicates the effect of the *A_ws_*, which is estimated by using Equation (10) and shown in Figure 9 as a function of the DS inlet hydraulic pressure.

From a comparison between the obtained *A_wm_* and *A_ws_* values, the controlling phase for water transfer can be predicted. It can be seen in the case of Experiment 1, where freshwater is used as FW and seawater as DS, that the membrane phase controls water transfer at low hydraulic pressures (*A_wm_* value is lower than that of *A_ws_*), while at higher hydraulic pressure, the solution phase appears to be the dominating one. In the cases of Experiments 2 and 3, where higher concentration solutions are used, *A_ws_* is lower than *A_wm_*, which reflects the higher resistance of the solution.

**Figure 10:**
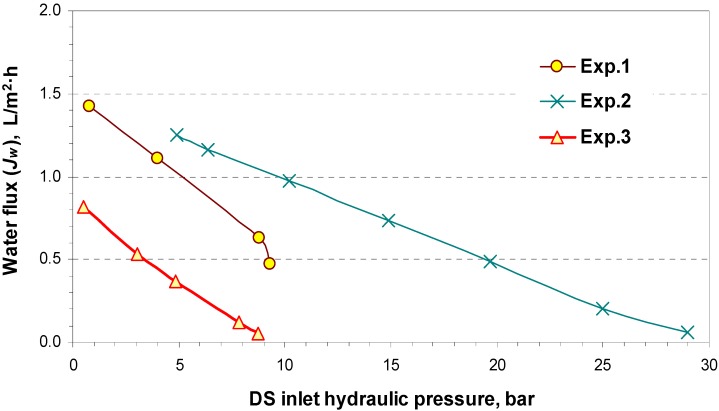
Water flux (*J_w_*) as a function of the DS inlet hydraulic pressure.

By using brackish water as FW with seawater DS (Exp.3), utilizing brackish water as FW and high salinity water as DS (Exp.2), and employing freshwater as FW with seawater DS (Exp.1), the obtained experimental value for water flux as a function of the draw solution pressure is illustrated in Figure 10 in a PRO set up.

As DS inlet hydraulic pressure increases, water flux decreases until it reaches zero due to the reduction of driving force (ΔП–Δ*P*). In addition, the results in Figure 10 show that water flux decreases by raising the concentration of feed water FW (Exp.3 compared with Exp.1), because the concentration polarization effect becomes more severe as the concentration of feed increases. However, when comparing water flux in Experiment 2 with that in Experiment 3, it can be seen that higher water flux is observed when the concentration of DS increases in a constant feed solution concentration.

**Figure 11:**
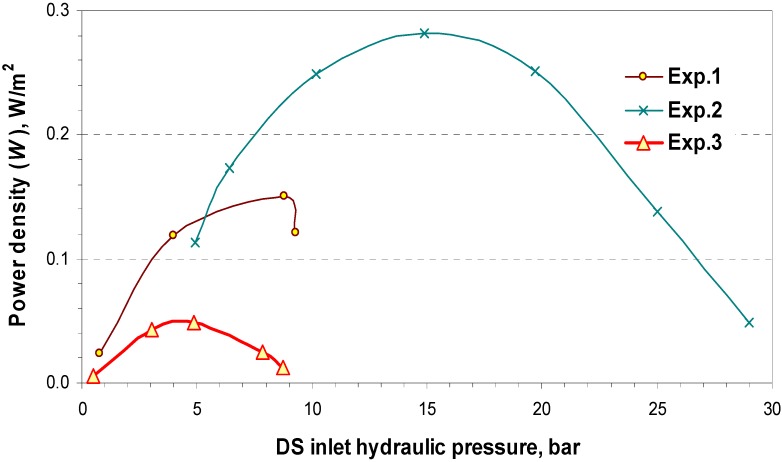
Power Density (*W*) as a function of the DS inlet hydraulic pressure for different osmotic systems.

Figure 11 illustrates the effect of the DS side hydraulic pressure on power density for the three experiment sets 1, 2 and 3. The produced power density increases by increasing the DS feed hydraulic pressure and reaches a maximum when the hydraulic pressure is approximately half of the hydraulic pressure at the zero water flux point, then it trends downward to zero.

Moreover, the power density (*W*) obtains a much higher value (up to 0.28 W/m^2^) when the higher concentration of the DS (Exp.2) is used compared with Experiment 3 due to the higher osmotic pressure difference ∆П_f_ as the driving force. However, with a constant concentration of DS and an increase of the feed concentration (Exp.1 and 3), power density decreases because of the reduced osmotic pressure difference. Therefore, power density has been found to be dependent on the inlet concentrations of the DS and the FW, because a higher chemical potential difference between DS and FW provides a large free energy capacity. The achieved power density 0.28 W/m^2^ shows a significant effect of concentration polarization, which reduces the attainable water flux *J_w_* and limits the efficiency to less than 1 W/m^2^. However, these results illustrated in Figure 11 were obtained for the specific membrane type utilized in the present study. Different figures can be expected when other membranes or modules are used (under similar operational conditions). Although based on the economical calculations by Statkraft, osmotic power will become financially feasible when membranes produce a minimum power density of 5 W/m^2^ [35].

## 5. Commercial Potential and Cost Estimation

The osmotic energy is a reliable energy source and has potential to generate a constant power to operate more than 8000 h per year continuously compared with other renewable sources of energy. The HOP technology employs techniques and equipment, such as membranes, pumps, energy recovery systems, and turbines, which are currently used in desalination, water treatment, and hydropower industries, making HOP application and development relatively straightforward. Additionally, the combination of osmotic power with a conventional RO desalination process, coupled with other renewable energy resources, such as solar, wind, and low-grade heat could result in substantially lower energy consumption and less reliance on fossil fuels. In fact, in certain conditions, the RO plant when incorporated with a HOP unit could produce a large percentage of the power needed for the plant itself. The competitiveness of the global market of osmotic energy compared to other renewable energy sources such as solar, wind (onshore and offshore), biomass, hydra running and dam, and also other non-renewable sources including Nuclear, gas, oil, and coal was analysed by Skilhagen in Statkraft Company, [35] based on the existing market prices for all sources in a large project. The results are given in Table 4 and show a bright future market for osmotic power [35]. The cost of osmotic power estimated by Statkraft predicts that an osmosis-power plant could produce eco-electricity for 50–100 €/MWh [8].

**Table 4:** The Estimated Energy Cost of Renewable and Non-Renewable Sources by 2030 [35]. Data from reference [35].

Renewable/Non-Renewable Energy Sources	Estimated Energy Cost (€/MWh) Forecast 2030
Nuclear	45
Gas CCGT	85
Oil CC	125
Coal PCC	80
Hydro dam	85
Hydro running	48
Biomass	88
Wind Offshore	115
Wind Onshore	90
Solar	160
Osmotic Energy	50–100

Research and development activities at the Centre of Osmosis Research and Application (CORA) at the University of Surrey [8,36] in collaboration with Modern Water plc have involved both pilot plant and theoretical studies to demonstrate the potential of hydro-osmotic power in practice. The estimated designs parameters in Section 4.1 for 25 MW net electricity production in a closed-cycle HOP plant have been applied with economic parameters to carry out the calculations of capital cost and the cost of electricity in this part. The electricity cost is the principle of the feasibility of the commercial utilization of osmotic power and results from the revenues and investment costs over the lifespan of the osmotic power plant. The investment costs including capital costs of the power plant, operational and maintenance costs, lifespan of the power plant, return-on-investment period, and depreciation time of the main infrastructure, interest rate and index ratios were estimated or calculated using the available data in our model. The revenues originating from the osmotic power production were foreseen to be constant throughout the lifespan of the power plant in this study. A return-on-investment period of 15 years was assumed and an annual cost of operation and maintenance was considered to be equal to 3% of the total capital costs of the power plant. The regeneration unit was assumed to be similar to other osmotic (FO) units with similar membrane permeability. The input economic parameters in this study are listed in Table 5.

**Table 5:** Assumed input economic parameters (the cost here is in GBP Sterling).

**Plant Economic Performance**	**Value**
Discount Rate (%)	6
Plant Lifetime (year)	15
**Energy**	**Value**
Industrial Electricity Price (£/kWh)	0.05
**Turbine and Generator**	**Value**
Specific Investment Cost (£/MWe)	200,000
O&M (% capital)	3
Life time (year)	15
Maximum Unit Capacity (MWe)	100
**Low pressure pumps**	**Value**
Specific Investment Cost (£/MWe)	10,000
Life time (year)	15
O & M (% capital)	2.5
Maximum Pump Unit Capacity (MWe)	100
**Pressure exchanger system (PES)**	**Value**
Specific Investment Cost (£/MW)	50,000
O&M costs (% capital)	2.5
Life time (year)	15
Maximum Unit Capacity (MWe)	5
**Membranes (for both OMU and RU)**	**Value**
Specific Investment Cost (£/module)	70
Life time (year)	7
Max Module Area (m^2^)	100.8
**Pipes and valves**	**Value**
Specific Cost (£/km)	100,000
Life time (year)	15
O&M costs (% capital)	2.5
Specific inv. Cost of Brine pre-treatment (% plant capital)	4
control system (£/MWe)	10,000
Specific cost of Draw Solution(£/m^3^)	10
Plant Availability (%)	90
Yearly Salary of Personnel (£/year)	25,000
No. of Personnel	66

**Figure 12:**
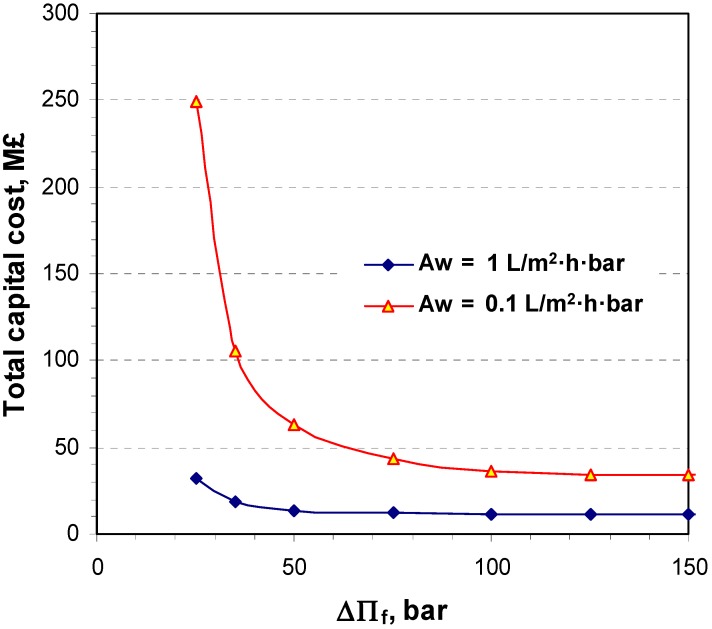
The total estimated capital cost of a closed cycle HOP plant for 25 MW net power production as a function of the osmotic pressure differnces (∆П_f_) at the OMU utilizing a 15 bar hydraulic pressure at the DS side.

For two different system permeabilities (*A_w_*), 0.1 and 1 L/m^2^·h·bar, the total capital cost of a closed cycle plant (as illustrated in Figure 2) for 25 MW net electricity production was calculated as a function of the osmotic pressure differences, ∆П_f_, between the inlet concentrated DS and the inlet diluted FW to the osmotic membreane unit, OMU. As can be seen from Figure 12, the membrane with high permeability has a sufficient impact on the reduction of capital cost and thus a larger impact on the energy unit cost.

**Figure 13:**
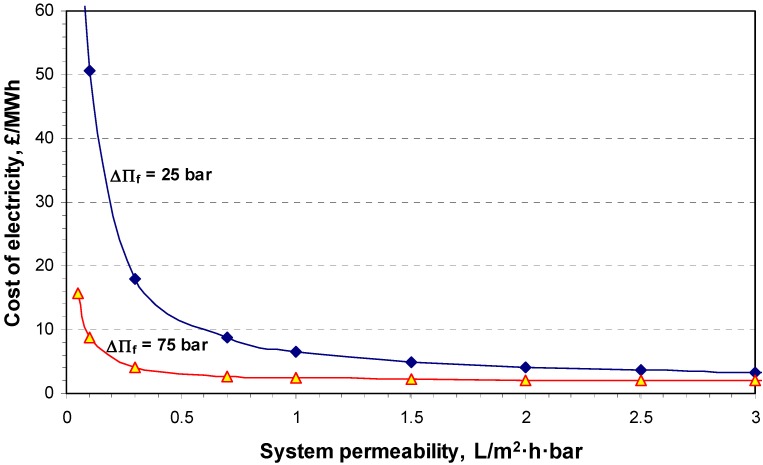
The estimated cost of electricity of the proposed closed cycle HOP plant for 25 MW net power production at two osmotic pressure differences (∆П_f_) at the FO unit, 25 and 75 bar, utilizing a 15 bar hydraulic pressure at the DS side, as a function of the system permeability.

The results presented in Figure 12 could lead to the conclusion that a substantial reduction of total capital cost needs a membrane as thin as possible to utilize the osmotic pressure difference while withstanding a pressure difference greater than 25 bar. The unit energy cost produced by osmostic power was then estimated for 25 MW net power production at two osmotic pressure differnces (∆П_f_) at the FO unit, 25 and 75 bar, by utilizing a 15 bar hydraulic pressure at the DS side, as a function of the system permeability (Figure 13).

The results suggest a projected cost of £30/MWh of electricity for an osmotic pressure difference higher than 25 bar and indicate that increasing the system permeability by another 0.3 L/m^2^·h·bar has little or no effect on the overall cost of the produced electricity. These values demonstrate that osmotic power could be financially feasible compared to the levelized cost for alternative renewable energy sources; however an improvement in membrane permeability and subsequently power density is recommended to decrease the total capital cost of an HOP plant. Today, the capital cost of a commercial osmotic power plant would be extremely high as the concentration polarization phenomenon limits the efficiency of the membranes necessary for a large membrane area to overcome the low power density. Therefore, a significant improvement of the design and production of a high semi-permeable membrane able to withstand high pressure differentials is needed for the production of osmotic power on a commercial basis.

## 6. Conclusions

In this study, both theoretical and experimental investigations of the potential of osmotic energy using a salinity gradient for power generation were carried out. The results indicate a high potential of osmotic energy for power generation using the HOP Process. Several theoretical calculations have been presented, which show e.g., that clean electricity could be produced using this process at a projected cost of £30/MWh, if a suitable membrane is used, and the osmotic potential difference between the two solutions is greater than 25 bar; a condition that can be readily achieved in many sites around the world. The results also illustrate the effect of the system permeability to water and the osmotic pressure difference across the membrane at the osmotic membrane unit on the HOP plant cost and productivity. This study further presents pilot plant results under different operational conditions. The experiments show the effect of physical properties of the feed water and the draw solutions on the water permeability across the semi-permeable membrane in PRO processes. The water permeability is a critical issue when the HOP process feasibility is being evaluated. Increase in the membrane permeability decreases the capital cost and increases the power productivity. The interaction between the fluid properties and the membrane properties needs to be considered when these processes are to be developed in the future.

## Nomenclature

### Abbreviations

HOP: Hydro-Osmotic Power
RE: Renewable Energy
RO: Reverse Osmosis
SEC: Specific Energy Consumption
ERS: Energy Recovery System
PRO: Pressure Retarded Osmosis
PES: Pressure Exchange System
OE: osmotic energy
FO: Forward Osmosis
DS: Draw Solution
CTA: Cellulose Triacetate
R&D: Research & Development
MW: Modern Water
CORA: Centre for Osmosis Research and Applications
OMU: Osmotic Membrane Unit
FW: Feed Water
RU: Regeneration Unit
OHE: Osmotic Heat Engine
SDPFFR: Solution-Diffusion Pore-Flow Fluid-Resistance
ASDPF: Analytical Solution-Diffusion Pore-Flow

### Symbols

*J_w_*: Water flux (L/m^2^·h)
*A_W_*: System permeability to water (L/m^2^·h·bar)
∆П: Net osmotic pressure difference across membrane
∆*P*: Net hydraulic pressure difference
*W*: Density of power (W/m^2^)
*A_wm_*: The membrane phase permeability (L/m^2^·h·bar)
*A_ws_*: The solution phase, the DS and the FW, permeability (L/m^2^·h·bar)
δ*_mo_*: Membrane thickness
ε*_o_*: Membrane porosity
τ*_o_*: Membrane tortuosity
*d_mo_*: Membrane mean pore diameter
*D_wo_*: Self-diffusivity
ρ*_wo_*: Density
μ*_o_*: Viscosity
*M_w_*: Molecular weight of pure water at the reference temperature (*T_o_* = 298.15 K)
*R_g_*: Universal gas constant
T, Y, K, H: Constant values
П: Osmotic pressure (bar)
*P*: Hydraulic pressure (bar)
*Q*: The volumetric flow rate (m^3^/s)
*Q_w_*: The permeated water flow rate across the membrane (m^3^/s)
*P_G_*: The gross power production (MW)
*ρ_E_*: The energy density (kWh/m^3^)
*E_S_*: The specific energy production (kWh/m^3^)
